# Improved PEG-Based Construction of Analog Fountain Codes

**DOI:** 10.3390/e26100841

**Published:** 2024-10-05

**Authors:** Xue Li, Qin Huang

**Affiliations:** School of Electronic and Information Engineering, Beihang University, Beijing 100191, China; lixue19@buaa.edu.cn

**Keywords:** analog fountain codes, progressive edge-growth, weight coefficients

## Abstract

This paper proposes an improved *progressive edge-growth* (PEG) construction of analog fountain codes (AFCs). During edge selection, it simultaneously allocates weight coefficients in descending order. Analysis shows that our proposed construction reduces the probability of large weight coefficients involved in harmful short cycles. Simulation results indicate that it has good block error rate (BLER) in short block length regime.

## 1. Introduction

With the emergence of applications such as intelligent transportation, tele-surgery, and industrial automation, three main service categories are defined in the fifth generation (5G) mobile network. Among these, ultra-reliable low latency communication (URLLC) poses the most significant challenges due to its physical layer design [1]. URLLC requires a short end-to-end latency, along with high reliability evaluated by block error rate (BLER). These strict constraints impose stringent requirements for the design of channel coding in short block length regime.

Recently, analog fountain codes (AFCs) [2,3,4], a type of rateless codes, have shown their great potential to improve the latency and reliability while keeping bit-level granularity. Several impressive studies [5,6,7,8,9,10,11,12] have explored the coding schemes and constructions of AFCs to make them promising for URLLC. In [5], optimal AFC parameters were analyzed and designed to minimize the bit error rate. In [7], a low-complexity weight-adaptive AFC transmission scheme was proposed based on extrinsic information transfer (EXIT) analysis. In [9], Bose–Chaudhuri–Hocquenghem (BCH) precoders were designed for AFCs to improve performance for short packet communication. In [11], joint design of AFCs and quasi-gray constellation mapping modulation was proposed, approaching the benchmark. In [12], a short partitioned transmission strategy based on AFCs was proposed to further enhance the error rate and latency performance.

AFC generator matrices construction involves two steps: edge selection and weight coefficient allocation. In [2], the edges were selected to maximize the minimum variable node (VN) degree of AFCs. Weight coefficients were allocated randomly. In [6], short AFCs (S-AFCs) reduced error floors by processing these two steps according to a reliability measure. In [8], Online AFC selected edges and allocated weight coefficients according to feedback information, thereby enhancing reliability. Since short cycles deteriorate the performance of the BP decoding, the PEG algorithm [13] was proposed to reduce short cycles of low-density parity-check (LDPC) codes. In [10], an efficient coefficient progressive edge-growth (WC-PEG) algorithm avoided short cycles to obtain good performance. It selected weight coefficients in the same number of times in each column of generator matrix to avoid reducing the rank of generator matrix.

In this paper, we show that large weight coefficients have high mutual information; thus, they contribute most during the BP decoding. Therefore, it is important to avoid them in short cycles, which may severely deteriorate the decoding performance. Note that PEG maximizes the cycle length, and the earlier selected edges are less likely to be involved in short cycles. We propose to add edges and allocate their weight coefficients in descending order simultaneously. To be specific, we select edges with the smallest reliability measure, and allocate larger weight coefficients to earlier selected edges. Our analysis shows that the proposed construction effectively decreases the probability that larger weight coefficients are involved in short cycles. The simulation results show that it has higher reliability over the existing constructions in short block length regime.

This paper is organized as follows. Section 2 presents the necessary background. Section 3 discusses our proposed construction as well as the simulation results. Section 4 concludes the paper.

## 2. Analog Fountain Codes and the PEG Algorithm

In this section, encoding and decoding processes of AFCs are briefly introduced. Then, PEG applied to AFC construction is reviewed.

### 2.1. Preliminaries of AFC

Consider an information sequence $b^{\prime }$ comprising *k* information bits ${b^{\prime }}_{i}\in \left\{0,1\right\}$, where i=0,1,…,k−1. $b^{\prime }$ is binary phase shift keying (BPSK) modulated, resulting in $b=\left(b_{0},b_{1},\ldots ,b_{k-1}\right)$, where $b_{i}\in \left\{-1,1\right\}$. Each AFC codeword $c=\{c_{0},c_{1},\ldots ,c_{n-1}\}$ is then generated as

(1) $$c={\mathrm{Gb}}^{T}.$$

**Figure 1:**
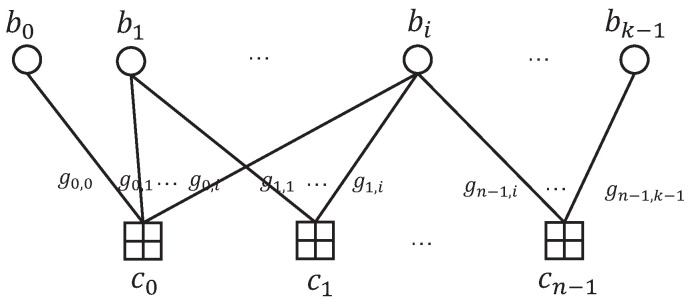
The Tanner graph of an AFC.

Here, G is the generator matrix:

(2) $$G=\left[\begin{array}{cccc} g_{0,0} & g_{0,1} & \cdots & g_{0,k-1}\\ g_{1,0} & g_{1,1} & \cdots & g_{1,k-1}\\ \vdots & \vdots & \ddots & \vdots \\ g_{n-1,0} & g_{n-1,1} & \cdots & g_{n-1,k-1} \end{array}\right]$$

and $b^{T}$ is the transpose of b. The corresponding Tanner graph is shown in Figure 1.

The degree *d* of AFC is defined as the number of nonzero elements in each row of **G**. The *d* nonzero elements correspond to a predefined weight set $W=\left\{w_{0},w_{1},\ldots ,w_{d-1}\right\}$, where w∈W is called the weight coefficient. Without loss of generality, we set $w_{0}>w_{1}>\cdots >w_{d-1}>0$.

After being transmitted through an additive white Gaussian noise (AWGN) channel, r=c+n is received, where **n** is the noise vector with zero mean and variance $\sigma^{2}$.

At the receiver side, a simplified compressive sensing belief propagation (CS-BP) decoder [14] is employed. The CS-BP decoder is a variant of the BP decoding which can be used in compressive sensing and AFC. The decoding process is briefly reviewed as follows. Let $\mu_{v\to c}\left(b_{i}\right)$ and $\mu_{c\to v}\left(b_{i}\right)$ denote the message transmitted from the VNs $b_{i}$ to check nodes (CNs) $c_{j}$ and vice versa, respectively. After a fixed number of iterations of $\mu_{v\to c(b_{i})}$ and $\mu_{c\to v}\left(b_{i}\right)$, the marginal distribution $f(b_{i})$ is obtained by

(3) $$f\left(b_{i}\right)=\prod_{j\in J_{i}}\mu_{u\to v}\left(b_{i}\right),$$

where $J_{i}$ denotes the set of CNs connected to $b_{i}$. Then, the decoder outputs the hard decision based on $f(b_{i})$. More details of the CS-BP decoder can be reached in [14].

### 2.2. The PEG Algorithm

Short cycles on Tanner graphs may deteriorate the decoding performance of the BP. Thus, the PEG [13] maximizes the cycle length in the Tanner graph by progressively establishing edges between VNs and CNs in an edge-by-edge manner. VN degree is defined as the number of nonzero elements in each column of G.

Suppose PEG is used to construct an n×k matrix G for AFC. For a given $c_{j}$, where 0≤j<n, we define its neighbor VNs within a depth *l* as $N_{c_{j}}^{l}$, and the remaining VNs are denoted by $\bar{N_{c_{j}}^{l}}$. If the subgraph from $c_{j}$ is fully expanded in depth *l*, two cases may happen. The cardinality of $N_{c_{j}}^{l}$ may stop increasing but $\left|N_{c_{j}}^{l}\right|<k$, or $\bar{N_{c_{j}}^{l}}\neq \emptyset$ but $\bar{N_{c_{j}}^{l+1}}=\emptyset$. Let $\left(c_{j},b_{i}\right)$ denote the edge connecting $c_{j}$ and $b_{i}$. At last, the newly established edge is $\left(c_{j},b_{i}\right)$, where $b_{i}$ is picked from the set $\bar{N_{c_{j}}^{l}}$ with the lowest VN degree. Note that length-*r* cycle arises if $\bar{N_{c_{j}}^{l+1}}=\emptyset$, where r=2(l+1).

When PEG is used to construct generator matrices for AFC, both edge selection and weight coefficients allocation should be considered. In [10], if the edge $\left(c_{j},b_{i}\right)$ is selected according to PEG, the weight coefficients are suggested to be allocated according to their appearance in the *i*-th column of G. Specifically, we calculate the times that each weight coefficient appears among the nonzero elements in the *i*-th column. The one with the lowest number of times should be allocated in order to balance the weight of columns in G.

## 3. Improved PEG-Based Construction

In this section, EXIT charts of different weight coefficients at CNs are shown. To avoid large weight coefficients involved in short cycles, we propose to add edges and allocate their weight coefficients simultaneously during edge selection. Analysis shows that the proposed construction efficiently reduces the probability of larger weight coefficients involved in the edges of short cycles.

### 3.1. EXIT Charts of Different Weight Coefficients

Different weight coefficients contribute differently in the decoding process at CNs. To illustrate that, EXIT charts for various weight coefficients are presented below.

Let $b=b_{0}\cup b_{1}\cup \cdots \cup b_{d-1}$ denote the information bits. Without loss of generality, for each CN $c_{j}$ connected to *d* VNs $b_{i_{0}},b_{i_{1}},\cdots ,b_{i_{d-1}}$, let $g_{i_{s},j}=w_{s}$, where s∈{0,1,⋯,d−1}. Then $b_{i_{s}}\in b_{s}$. In this way, information bits are divided into *d* parts, where bits in $b_{s}$ are connected to CN through an edge allocated with $w_{s}$.

Let $I_{A,C}$ and $I_{E_{s},C}$ denote the input mutual information and extrinsic mutual information of the *s*-th weight coefficient for CN, respectively. For each $b_{s}$, the mutual information between $b_{s}$ and its corresponding log-likelihood ratio (LLR) value $L_{s}$ computed at the CNs is represented as

(4) $$I_{E_{s},C}=\sum_{b_{s}\in \left\{-1,1\right\}}\int_{-\infty }^{+\infty }p_{E}\left(L_{s}|b_{s}\right)\log_{2}\frac{p_{E}\left(L_{s}|b_{s}\right)}{p_{E}\left(b_{s}\right)}dL_{s}.$$

Here, $p_{E}$ represents the conditional probability distribution function of $L_{s}$. As can be seen in Equation (4), each calculation of $I_{E_{s},C}$ is based on bits $b_{s}$ and LLR $L_{s}$. It means that after one decoding process at the CNs, CNs output extrinsic mutual information $I_{E_{s},C}$ to VNs which are connected through weight coefficients $w_{s}$. It is difficult to compute $p_{E}$ directly because of the convolution operation. Thus, the Monte Carlo methods are used to estimate $p_{E}$ for AFC [7]. Note that $L∼N(0,\sigma_{L})$, where $\sigma_{L}$ can be generated from $I_{A,C}$ as

(5) $$\sigma_{L}=J^{-1}\left(I_{A,C}\right).$$

The detailed definition of *J*-function can be reached in [15].

**Figure 2:**
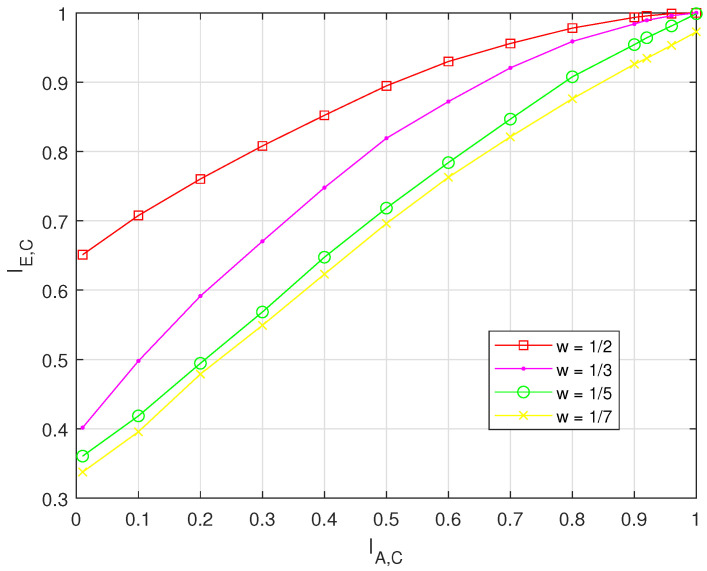
EXIT charts of different weight coefficients, where d=4, $W_{s}=\left\{1/2,1/3,1/5,1/7\right\}$, and signal–noise ratio (SNR)=18 dB.

Figure 2 shows the mutual information for four different weight coefficients of AFC, respectively. It can be seen that with the same $I_{A,C}$, the mutual information is larger when weight coefficient is larger. These results indicate that larger weight coefficients contribute higher in the BP decoding.

### 3.2. Improved PEG-Based Construction Method

It is well known that short cycles may deteriorate the performance of the BP decoding. Large weight coefficients should not be involved in harmful short cycles due to their high contribution in the BP decoding. AFC construction involves two steps: edge selection and weight coefficient allocation. From these two aspects, we propose to construct generator matrix as follows.

During edge selection, we balance the weight of columns in G with a reliability measure denoted by $\sum_{p=0}^{i-1}g_{p,q}^{2}$. The reliability measure represents the sum of squares of weight coefficients for the *q*-th column of G before the edges for $c_{i}$ are established. Apparently, if a column has a relatively low reliability measure, a corresponding edge should be selected to enlarge its reliability measure. Thus, each time the PEG establishes a candidate set of VN for edge selection, the set is further downsized based on the smallest reliability measure set $Q=\{q|\arg\min_{q}\sum_{p=0}^{i-1}g_{p,q}^{2}\}$.

With regard to weight coefficient allocation, recall that the PEG maximizes the cycle length, which means that the edges selected earlier are more likely to be in large cycle. In other words, the edges selected later are more likely to be in the short cycle. Thus, they should not be allocated with larger weight coefficients. It forms the weight coefficient allocation strategy that allocating larger weight coefficients to earlier selected edges.

The proposed construction is presented as follows and the details are summarized in Algorithm 1. Given the parameters *k*, *n*, *d*, and W, an edge-selection procedure is initiated. Let $E_{c_{i}}^{j}$ denote the *j*-th edge selected for $c_{i}$, where 0≤j<d. The placement of a new edge $E_{c_{i}}^{0}$ on the graph considers the reliability measure of each column. Specifically, $E_{c_{i}}^{0}\leftarrow \mathrm{edge}\left(c_{i},b_{q}\right)$, where *q* is randomly selected from $Q=\{q|\arg\min_{q}\sum_{p=0}^{i-1}g_{p,q}^{2}\}$. Subsequently, the largest weight coefficient $w_{0}$ is allocated to $E_{c_{i}}^{0}$ as $g_{i,q}=w_{0}$.

After that, the subgraph from $c_{i}$ is expanded using the strategy of PEG [13], ending up with the set $\bar{N_{c_{i}}^{l}}$. When the expanded stage is finished, $E_{c_{i}}^{j}\leftarrow \mathrm{edge}\left(c_{i},b_{q}\right)$, where *q* is randomly selected from a refreshed $Q=\{q|\arg\min_{q}\sum_{p=0}^{i-1}g_{p,q}^{2},b_{q}\in \bar{N_{c_{i}}^{l}}\}$. Then, the next weight coefficient $w_{j}$ is allocated to $E_{c_{i}}^{j}$ as $g_{i,q}=w_{j}$. This step is repeated until *d* edges are selected, and then the process moves to the next CN.
**Algorithm 1** Improved PEG-based construction algorithm.**Input:** *k*, *n*, *d*, W**Output:** AFC generator matrix G1:G=zeros(n,k)2:**for** i=0 to n−1 **do**3:   **for** j=0 to d−1 **do**4:     **if** j=0 **then**5:        $Q=\{q|\arg\min_{q}\sum_{p=0}^{i-1}g_{p,q}^{2}\}$6:        $E_{c_{i}}^{0}\leftarrow \mathrm{edge}\left(c_{i},b_{q}\right)$, where *q* is randomly selected from *Q*.7:        Update the $g_{i,q}=w_{0}$.8:     **else**9:        expand a subgraph from $c_{i}$ up to depth *l* under the current graph setting such that the cardinality $N_{c_{j}}^{l}$ stops increasing but $\left|N_{c_{i}}^{l}\right|<k$, or $\bar{N_{c_{i}}^{l}}\neq \emptyset$ but $\bar{N_{c_{i}}^{l+1}}=\emptyset$.10:        $Q=\{q|\arg\min_{q}\sum_{p=0}^{i-1}g_{p,q}^{2},b_{q}\in \bar{N_{c_{i}}^{l}}\}$11:        $E_{c_{i}}^{j}\leftarrow \mathrm{edge}\left(c_{i},b_{q}\right)$, where *q* is randomly selected from *Q*.12:        Update the $g_{i,q}=w_{j}$.13:     **end if**14:   **end for**15:**end for**16:Return G.

### 3.3. Analysis of Improved PEG-Based Construction

Suppose that after applying Algorithm 1, a new initialized CN $c_{n}$ introduces length-*r* cycles following the selection of $E_{c_{n}}^{d-e}$, where *e* denotes the minimum number of length-*r* cycles introduced by $c_{n}$. During the selection of $E_{c_{n}}^{a}$, where d−e≤a<d, *a* selected edges $E_{c_{n}}^{0},E_{c_{n}}^{1},\cdots ,E_{c_{n}}^{a-1}$ during the selection of $c_{n}$, and nd edges established before the initialization of $c_{n}$ are candidates that can be randomly connected to form a length-*r* cycle.

Denote the r−2 edges of the length-*r* cycle established before the initialization of $c_{n}$ as $\left(c_{j_{0}},b_{i_{0}}\right),\left(c_{j_{1}},b_{i_{1}}\right),\cdots ,\left(c_{j_{r-3}},b_{i_{r-3}}\right)$, where 0≤j<n and 0≤i<k. Without loss of generality, denote $E_{c_{n}}^{a}\leftarrow \left(c_{n},b_{i_{0}}\right)$ as the edge closes the cycle, and denote $E_{c_{n}}^{a^{\prime }}\leftarrow \left(c_{n},b_{i_{1}}\right)$ as the edge forming the cycle due to the presence of $\left(c_{n},b_{i_{0}}\right)$, where $0\leq a^{\prime }<a$.

Moreover, divide W into two subsets according to $E_{c_{n}}^{d-e}$ as $W=W_{\mathrm{large}}\cup W_{\mathrm{small}}$, where $W_{\mathrm{large}}=\left\{w_{0},w_{1},\cdots ,w_{d-e-1}\right\}$ and $W_{\mathrm{small}}=\left\{w_{d-e},w_{d-e+2},\cdots ,w_{d-1}\right\}$ represent the sets consisting of relatively large and small weight coefficients, respectively. Based on the aforementioned discussion, the probabilities that a weight coefficient involved in the above edges are analyzed case by case.

*Case 1:*

(6) $$P(g_{j,i}=w)=1/d,\;w\in W,$$

where $\left(c_{j},b_{i}\right)$ is one of the r−2 edges. The weight coefficient in $\left(c_{j},b_{i}\right)$ is allocated before the PEG selects edges for $c_{n}$. Thus, it is considered as randomly selected from W.

*Case 2:*

(7) $$P\left(g_{n,i_{0}}=w\right)=0,\;w\in W_{\mathrm{large}}.$$

Considering that length-*r* cycles arise after selecting $E_{c_{n}}^{d-e}$, and the weight coefficients are allocated in descending order, the last selected *e* edges for $c_{n}$ are allocated with $w\in W_{\mathrm{small}}$. On the contrary, any $w\in W_{\mathrm{large}}$ cannot be allocated to $g_{n,i_{0}}$.

*Case 3:*

(8) $$P\left(g_{n,i_{1}}=w\right)=\frac{1}{a}\leq \frac{1}{d-e},\;w\in W_{\mathrm{large}}.$$

The corresponding $\left(c_{n},b_{i_{1}}\right)$ is randomly connected from the *a* selected edges. Thus, *w* can be considered as randomly selected from $\left\{w_{0},w_{1},\cdots ,w_{a-1}\right\}$.

Based on the above analysis, r−2 edges $\left(c_{j},b_{i}\right)$, 1 edge $\left(c_{n},b_{i_{0}}\right)$, and 1 edge $\left(c_{n},b_{i_{1}}\right)$ have the probabilities in cases 1, 2, and 3, respectively. The probability that *w* is involved in an edge of the new introduced length-*r* cycles when $w\in W_{\mathrm{large}}$ is

(9) $$P\left(g_{j,i}=w\right)\leq \left(\frac{1}{d-e}+\frac{r-2}{d}\right)/r,\;w\in W_{\mathrm{large}},$$

where 0≤j≤n.

**Example** **1.** *Let us consider a 256×64 matrix G with degree d=4, which is generated using Algorithm 1. According to the parameter settings and the bound analysis in [10], a few length-4 cycles arise. Thus, we consider r=4 and e=1. W is divided into $W_{\mathrm{large}}=\left\{w_{0},w_{1},w_{2}\right\}$ and $W_{\mathrm{small}}=\left\{w_{3}\right\}$. Substitute d, e, and r into (9), $P(g_{j,i}=w)\leq 0.208$, when $w\in W_{\mathrm{large}}$. Through statistical analysis of a generated G, there are 25 length-4 cycles. The probabilities of weight coefficients involved in the length-4 cycles are shown in Table 1. These results verify our analysis for length-4 cycles.*

**Example** **2.** *Let us consider a 150×128 matrix G with d=4, which is generated using Algorithm 1. Similarly, we consider e=1 and r=6 for G. W is divided in the same way as in Example 1. Substitute d, e, and r into (9), $P(g_{j,i}=w)\leq 0.22$, when $w\in W_{\mathrm{large}}$. Through statistical analysis of a generated G, there are 28 length-6 cycles. The probabilities of weight coefficients involved in the length-6 cycles are shown in Table 2. These results verify our analysis for length-6 cycles.*

### 3.4. Complexity Analysis

The complexity of WC-PEG is O(nk) [13] in the worst case, mainly from expanding the Tanner graph. In addition to expanding the Tanner graph, our proposed algorithm involves calculating the reliability measure. It calculates *d* weight coefficients introduced by the last CN with complexity O(nd). Then, it sorts the *d* changed reliability measure with the remainder by binary insertion with complexity O(ndlogk). Thus, its overall complexity is O(n(k+dlogk)) in the worst case. All of these algorithms take moderate complexity for constructing generator matrices, which are designed offline.

### 3.5. Simulation and Discussion

In this subsection, we present the numerical results of the proposed construction and the constructions in the references. Here, we select d=8 and $W=\left\{1/2,1/3,1/5,\right.$$\left.1/7,1/11,1/13,1/17,1/19\right\}$ for AFC constructions, which are designed in [2]. In addition, d=4 and $W=\left\{1/2,1/3,1/5,1/7\right\}$ are also considered. Various construction methods such as random AFC, S-AFC, and WC-PEG are included as comparison. The proposed construction is labeled as IM-PEG. These settings are in line with the previous references. The CS-BP decoder with a maximum iteration number of 15 is applied in the receiver side.

**Figure 3:**
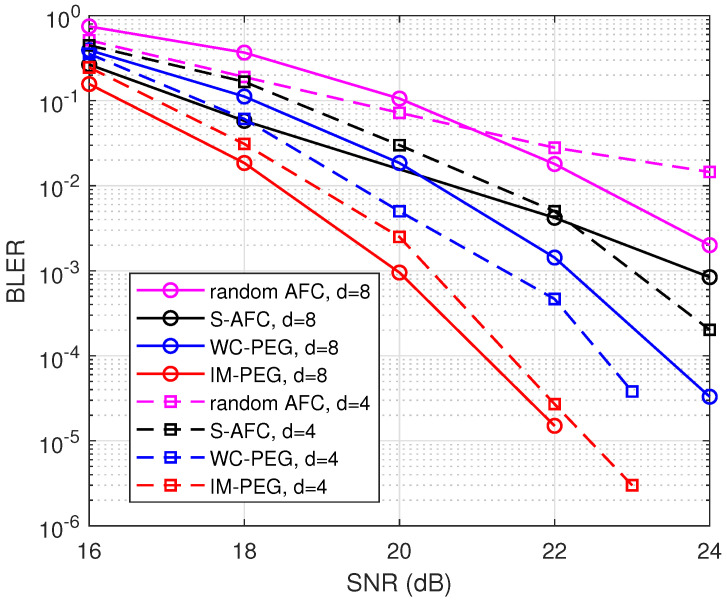
BLER of different AFC constructions with d=4 and d=8.

First, we simulate the BLER of different AFC constructions including random AFC, S-AFC, WC-PEG, and the proposed construction. Let R=k/n denote the code rate. Simulations are conducted with fixed rate R=2 and k=128. Various degrees d=4 and d=8 are considered, respectively. The AWGN channel with zero mean and variance $\delta^{2}$ is employed for the transmission of AFC codewords. Signal–noise ratios (SNRs) range from 16 dB to 24 dB. All of the parameters are set according to [2,6]. Please note that *R* can be larger than 1, since one AFC symbol involves *d* bits. It can be seen in Figure 3 that the improved PEG construction has good BLER performance. For example, when d=4, the proposed construction offers about 1 dB gain when BLER is lower than $10^{-4}$ over WC-PEG and S-AFC.

**Figure 4:**
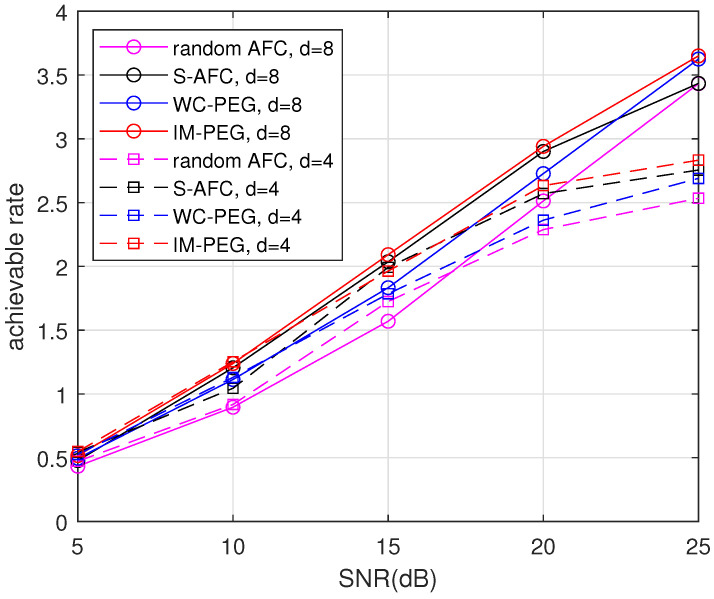
Achievable rates of different AFC constructions with d=4 and d=8.

Then, the achievable rates of different AFC constructions with k=128 are simulated. Similarly, various degrees d=4 and d=8 are considered, respectively. Figure 4 shows that the rates of the proposed construction are higher than others in a large range of SNRs, in both d=4 and d=8.

**Figure 5:**
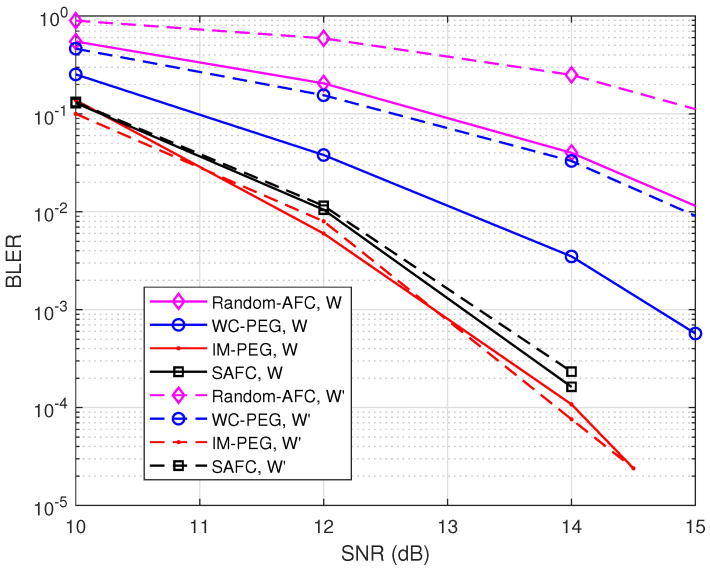
BLER of different weight sets with d=4 and R=1.

Moreover, BLER performance with various weight sets is presented in Figure 5. k=128, d=4, and R=1 are set for the simulation. $W^{\prime }=\left\{0.8670,0.432,0.2155,0.1073\right\}$ [6] is selected for comparison. It can be seen from Figure 5 that the proposed construction provides good error performance with $W^{\prime }$ as well.

## 4. Conclusions

In this paper, an improved PEG-based construction of AFC is proposed to enhance decoding performance. Specifically, we propose to allocate weight coefficients in descending order simultaneously when PEG selects edges for AFC, avoiding large weight coefficients involved in the short cycles. The analysis demonstrates that the proposed construction efficiently reduces the probability that larger weight coefficients are involved in short cycles. The simulation results show that it has good BLER performance and achievable rates.

## Figures and Tables

**Table 1:** Probabilities of weight coefficients involved in the length-4 cycles.

Weight Coefficient	Probability
$w_{0}$	20%
$w_{1}$	23%
$w_{2}$	18%
$w_{3}$	39%

**Table 2:** Probabilities of weight coefficients involved in the length-6 cycles.

Weight Coefficient	Probability
$w_{0}$	19%
$w_{1}$	21.4%
$w_{2}$	21.4%
$w_{3}$	38.2%

## Data Availability

The original contributions presented in the study are included in the article, further inquiries can be directed to the corresponding author.
